# On the Action of Atrophine in Painful Affections of the Face

**Published:** 1848-10

**Authors:** W. Philpot Brookes

**Affiliations:** surgeon to the Cheltenham General Hospital and Dispensary


### Collectanea.

*On the Action of Athrophine in Painful Affections of the Face, etc.* by W. Philpot Brookes, M. D., M. R. C. S. E., surgeon to the Cheltenham General Hospital and Dispensary :—A few weeks back, I was called in to a lady in this town, suffering from a severe cold, accompanied with a most intense and painful affection of the right side of the face, forehead, and around the orbit of the eye. The pain continued after all the symptoms of the cold had left her, and I could not allay it with warm fomentations or other common remedies. I at last tried the application of an ointment, composed of atrophine, five grains, lard, three drachms, with one drop of ottar of rose; a piece the size of a pea to be applied three times a day. The pain was allayed after the second application by day, but at night returned with as much violence as before. The remedy was continued, and after two days, all pain ceased, and has not since returned. The effect of it was so marked, that I am inclined to think it will prove a most useful remedy in painful neuralgic affections.

I must also mention the marked effect it had on the pupils of the eye, in this case, after the second application of it: they were dilated to a great extent, (much more than I ever saw from any other preparation of belladonna,) and continued so for two or three days after it was discontinued.

I have since tried it in the case of a man on whom I operated for cataract in both eyes. The one in the right eye was not perfectly depressed, and rose again; (he had also lippitudo of the eyelids from a burn.) Belladonna had but little effect on this eye, (although it perfectly dilated the other,) but the ointment of atrophine, three grains to two drachms, dilated it effectually. In a case of glaucoma I have now under treatment, belladonna will not increase the size of the pupil in either eye to any great extent, but the ointment does so satisfactorily.

I certainly think this active principle is well worthy of a trial; and it will be found a much cleaner, more certain and elegant form than any other preparation of belladonna.—*London Lancet.*

